# Acute Coronary Syndromes in Chronic Kidney Disease: Clinical and Therapeutic Characteristics

**DOI:** 10.3390/medicina56030118

**Published:** 2020-03-08

**Authors:** Mădălina Ioana Moisi, Marius Rus, Simona Bungau, Dana Carmen Zaha, Diana Uivarosan, Ovidiu Fratila, Delia Mirela Tit, Laura Endres, Delia Carmen Nistor-Cseppento, Mircea Ioachim Popescu

**Affiliations:** 1 Department of Preclinical Disciplines, Faculty of Medicine and Pharmacy, University of Oradea, 410073 Oradea, Romania; mada_vidican@yahoo.ro (M.I.M.); danaczaha@gmail.com (D.C.Z.); diana.uivarosan@gmail.com (D.U.); 2 Department of Medical Disciplines, Faculty of Medicine and Pharmacy, University of Oradea, 410073 Oradea, Romania; rusmariusr@yahoo.com (M.R.); ovidiufr@yahoo.co.uk (O.F.); procardia_oradea@yahoo.com (M.I.P.); 3 Department of Pharmacy, Faculty of Medicine and Pharmacy, University of Oradea, 410028 Oradea, Romania; mirela_tit@yahoo.com; 4 Department of Psycho-neurosciences and Recovery, Faculty of Medicine and Pharmacy, University of Oradea, 410073 Oradea, Romania; laura_endres@yahoo.com (L.E.); delia_cseppento@yahoo.com (D.C.N.-C.)

**Keywords:** acute coronary syndromes, chronic kidney disease, biomarkers, NT-proBNP, echocardiography

## Abstract

*Background and Objectives:* This study evaluated the clinical characteristics of the acute coronary syndromes (ACS) in chronic kidney disease (CKD) patients and established prognostic values of the biomarkers and echocardiography. *Materials and Methods:* 273 patients admitted to the cardiology department of the Clinical County Emergency Hospital of Oradea, Romania, with ACS diagnosis were studied. Two study groups were formed according to the presence of CKD (137 patients with ACS + CKD and 136 with ACS without CKD). Kidney Disease: Improving Global Outcomes (KDIGO) threshold was used to assess the stages of CKD. *Results:* Data regarding the medical history, laboratory findings, biomarkers, echocardiography, and coronary angiography were analysed for both groups. ACS parameters were represented by ST-segment elevation myocardial infarction (STEMI), which revealed a greater incidence in subjects without CKD (43.88%); non-ST-segment elevation myocardial infarction (NSTEMI), characteristic for the CKD group (28.47%, with statistically significance *p* = 0.04); unstable angina and myocardial infarction with nonobstructive coronary arteries (MINOCA). Diabetes mellitus, chronic heart failure, previous stroke, and chronic coronary syndrome were more prevalent in the ACS + CKD group (56.93%, *p* < 0.01; 41.61%, *p* < 0.01; 18.25%, *p* < 0.01; 45.26%, *p* < 0.01). N-terminal pro b-type natriuretic peptide (NT-proBNP) was statistically higher (*p* < 0.01) in patients with CKD; Killip class 3 was evidenced more frequently in the same group (*p* < 0.01). Single-vessel coronary artery disease (CAD) was statistically more frequent in the ACS without CKD group (29.41%, *p* < 0.01) and three-vessel CAD or left main coronary artery disease (LMCA) were found more often in the ACS + CKD group (27.01%, 14.6%). *Conclusions:* Extension of the CAD in CKD subjects revealed an increased prevalence of the proximal CAD, and the involvement of various coronary arteries is characteristic in these patients. Biomarkers and echocardiographic elements can outline the evolution and outcomes of ACS in CKD patients.

## 1. Introduction

The risk of coronary artery disease (CAD) in patients with chronic kidney disease (CKD) is comparable with the risk of CAD in patients with traditional risk factors. The association of the classic cardiovascular risk factor and the specific uraemia-related risk factors accelerates the atherosclerosis process, explaining the increased incidence of the major adverse cardiac events (which is the main cause of death in CKD subjects) [1,2]. Non-traditional risk factors (inflammation, proteinuria, anaemia, alterations in calcium and phosphate metabolism, oxidative stress) show an increased incidence as the kidney function declines [3]. Dyslipidaemia has an important role in the initiation of the atherosclerotic phenomenon, however, some important trials such as AURORA (a study to evaluate the use of rosuvastatin in subjects on regular haemodialysis: An Assessment of Survival and Cardiovascular Events) [4] and the 4D study (the German Diabetes and Dialysis Study–in German: Die Deutsche Diabetes Dialyse Studie) [5] revealed that statins did not improve the outcome of the patients with CKD, highlighting the fact that the disturbance of the lipid fractions is not elementary in the progression of the atheromatous plaque in subjects with impaired renal function [6]. Statins should represent an option in the early phases of the renal function deterioration, when the effect of the drugs is optimal [7].

Acute coronary syndromes (ACS) encompass non-ST-segment elevation myocardial infarction (NSTEMI), unstable angina, ST-segment elevation myocardial infarction (STEMI), and myocardial infarction with nonobstructive coronary arteries (MINOCA) [8]. In CKD patients, the presentation is atypical, and the main symptom is dyspnoea instead of the thoracic pain, and asymptomatic forms are usually detected in these patients [9].

The impaired renal function interferes with the excretion of the cardiac necrosis biomarkers—the cardiac troponins (cTn), making the diagnosis of the ACS challenging. Myocardial infarction is defined as elevation of the cardiac necrosis biomarkers above the 99th percentile myocardial infarction of the healthy reference population, corroborated with specific chest pain and electrocardiographic signs of cardiac ischemia [10]. Guidelines elaborated by the European Society of Cardiology (ESC) recommend serial measurements of the necrosis enzymes, proposing an algorithm that requires initial determination of the cTn followed by new assessments at 1 or 3 h [10].

An important aspect, the renal nihilism, illustrates a predisposition for choosing medical management in subjects with impaired kidney function instead of the interventional or surgical myocardial revascularization. The outcome of the ACS in CKD subjects is negatively influenced by the mentioned therapeutic attitude and the underuse of appropriate doses of antiplatelet therapy or thrombolytic therapy explaining the increased rate of these patients’ cardiovascular mortality [11]. The treatment of ACS in patients with CKD has many issues. Thus far, there is no optimal treatment strategy for this type of patients. There are questions and concerns on the treatment for CKD patients in the incipient phases of STEMI and about using the aggressive reperfusion strategy (which implies both fibrinolytic therapy and primary coronary revascularization procedure). In patients with STEMI, the trials have shown the effect of thrombolytic agents in reducing mortality, but in many of them, analysis was not performed for patients with CKD [12,13]. The risk of bleeding, especially the increased incidence of intracranial haemorrhage, constitutes a reason for the less frequent use of thrombolysis in patients with CKD. However, primary percutaneous coronary intervention (PCI) may represent a favourable alternative therapy. A study conducted by Hobbach et al. revealed that the presence of CKD in a light to moderate form at the onset of STEMI determines a higher mortality rate in spite of using adequate therapy, but it also indicates the benefits of timely PCI for these patients [14]. Though the positive effect of pharmacological and mechanical coronary reperfusion in STEMI was proven, the optimum treatment scheme for STEMI patients also having CKD is still in discussion. Opinions about timely invasive scheme being indicated for patients with light CKD rather than behind time actions tended to fade away as the renal function deteriorated [15].

The coronary angiography followed by either PCI or coronary artery bypass grafting (CABG) should represent the treatment of choice in ACS irrespective of the CKD stage. A study that consisted in a five-year follow-up of the patients from the Syntax trial revealed the fact that patients with CKD undergoing PCI have a higher rate of negative outcome than subjects with impaired renal function undergoing CABG, especially in the subgroup of subjects with diabetes mellitus and extensive CAD [16].

The present study aimed to evaluate the clinical features of ACS in patients with CKD in north-western Romania and to highlight the evolutionary particularities of these patients, in order to establish an optimal case management in the emergency department. Predictors of unfavourable evolution were also relevant, in order to apply an immediate myocardial revascularization even in the case of those diagnosed with NSTEMI.

## 2. Materials and Methods

### 2.1. Study Design

A prospective study was performed, including 273 patients admitted with the diagnosis of ACS in the Cardiology department of the Clinical County Emergency Hospital of Oradea, Oradea, Romania, between 1 July 2018 and 1 September 2019. Inclusion criteria were as follows: new evidences of myocardial ischemia on the electrocardiography (ECG), with or without elevation of the myocardial necrosis enzymes as high-sensitivity troponins (hs-cTn); presence of typical angina or dyspnoea that could be interpreted as an angina-equivalent or asymptomatic cases with signs of myocardial ischemia; normal or decreased glomerular filtration rate (GFR). Exclusion criteria were represented by previous ECG changes consistent with myocardial ischemia without new signs of acute coronary disease; myocardial injury described as nonspecific elevation of cardiac necrosis enzymes in other conditions (like sepsis); advanced heart failure, chronic kidney disease, cerebrovascular accidents, myocardial trauma, or decreased value of the GFR due to dehydration and no other signs of kidney function alterations [10].

The study lot was divided into two groups depending on the presence of normal kidney function: the reference group was represented by 137 subjects with ACS+CKD, and the control group included 136 patients with ACS and normal renal function.

The entire research study was conducted respecting the World Medical Association Declaration of Helsinki (Ethical Principles for Medical Research Involving Human Subjects); in addition, it was approved by the Ethics Committee of the Clinical County Emergency Hospital of Oradea, Bihor County, Romania (decision no. 30088/10 December 2019). Each patient included in the study signed the informed consent form.

### 2.2. Methodology

The GFR was calculated using the Chronic Kidney Disease Epidemiology Collaboration (CKD-EPI) creatinine equation, the most indicated formula to correctly assess renal function [17]. An impaired renal function was defined, according to the National Kidney Foundation, as a persistent decreased GFR, more than 60 mL/min/1.73 m^2^, for more than 3 months, or kidney damage for more than 3 months with or without alterations of the GFR, involving either functional or morphological abnormal changes of the kidneys [17]. The Kidney Disease: Improving Global Outcomes (KDIGO) was used to characterize the CKD subjects, and the five stages [17] were described according to the GFR decrease (Table 1):

Unstable angina was defined as the presence of typical thoracic pain corroborated with new electrocardiographic changes and normal cardiac necrosis enzymes [7]. NSTEMI involves angina at onset, associated with more than 0.1 mv ST-segment depression on the ECG or the presence of new negative T waves in the context of elevated cardiac necrosis enzymes [7]. STEMI illustrates elevation of the ST-segment in two contiguous leads, detected in subjects with intense thoracic pain and elevated cTn. In men older than 40 years, the ST-segment elevation should be 2 mm in the precordial leads V2-V3 and more than 2.5 mm in male subjects younger than 40 years old [18]. In female subjects, the ST-segment elevation should reach at least 1.5 mm irrespective of the leads [18]. All the other leads require a ST-segment elevation more than 1 mm to fulfil the diagnosis [18]. The Sgarbossa’s criteria are specific for STEMI diagnosis in patients with left bundle branch block or ventricular paced rhythm and express a score formed from several criteria such as concordant ST-segment depression more than 1 mm in V1-V3 leads, concordant ST-segment elevation more than 1 mm in leads with a positive in large QRS complex (>0.12 seconds), and discordant ST-segment elevation more than 5 mm in leads with a negative QRS complex [18].

The newest concept is the MINOCA syndrome, which represents myocardial infarction with no significant lesions revealed on the angiography, and thus the stenosis of the coronary arteries lumen will be less than 50%. Some specific conditions such as myocarditis and Tako-Tsubo syndrome should be excluded before establishing the diagnosis of MINOCA [10].

Killip–Kimball classification was used to assess the left ventricular (LV) performance in patients with STEMI at admission. The four classes describe the degree of the pulmonary congestion as a response to the LV disfunction. Cardiogenic shock represents the last class and expresses the presence of haemodynamic instability [19].

The severity of shortness of breath was defined using the New York Heart Association (NYHA) classification of heart failure (Table 2) [20].

Subjects with previous chronic coronary syndrome were included in the study lot and the thoracic pain was described using the Canadian Cardiovascular Society grading of angina [13]. First stage showed no thoracic pain during normal activity and the other stages emphasized specific angina at normal activity. Fourth stage expressed angina at rest and reduced ability to perform ordinary activities [21].

The performance of the LV was appreciated according to the ejection fraction (EF), calculated using the Simpson’s method, the recommended formula specified in the guidelines elaborated by the American Society of Echocardiography and the European Association of Cardiovascular Imaging, corroborated with the value of N-terminal pro b-type natriuretic peptide (NT-proBNP), a product synthetized due to peripheral congestion [22]. NT-proBNP, an immunochemiluminescent assay using two polyclonal antibodies in a sandwich test format, was determined on the Pathfast automated analyser (Sysmex Nederlands) [23].

Urea and uric acid were spectrophotometrically measured using the Architect c4000 analyser (Abbott, Germany). The values of urea were defined as normal if they were between 15–55 mg/dL. In the case of uric acid dosing, the values that were within the limits of 3.5–7.2 mg/dL were considered appropriate. C-reactive protein (CRP) was assayed using the turbidimetric method, from patients’ sera, using the same analyser as that used previously; the values of this biomarker (specific for inflammatory syndrome) <0.50 mg/dL were considered normal.

Urgent coronary angiography was performed in patients with STEMI and suitable coronary anatomy, amendable by the procedure at admission or in less than 2 h from presentation, whereas subjects with multi-vessel CAD or significant left main stenosis were treated according to the ESC guidelines using surgical myocardial revascularization (CABG) [24].

### 2.3. Statistical Analysis

The database was gathered in a Microsoft Excel document. The statistical analysis was made using the Biostat Programme. The Kolmogorov–Smirnov test was applied to determine the data distribution. For the comparison of variables that were not normally distributed, the Mann–Whitney test was used. A value of *p* < 0.05 was considered statistically significant. When comparing the prevalence of a certain element in the two groups, a chi-squared test was applied, considering α = 0.05 as the confidence level.

## 3. Results

There were no statistically significant differences regarding the gender (*p* = 0.76) among the reference and the control lot. The only statistically significant demographical elements were represented by the mean age (*p* < 0.01), with a high mean age of 68.62 in the reference lot and the environment of origin (urban or rural) (*p* < 0.01), with the urban being more important in the reference lot. Comparison of demographic characteristics inside the same group showed that the masculine gender developed ACS much more frequently in both CKD groups and the normal kidney function group (*p* < 0.01) (Table 3). Staging of CKD in the ACS with CKD subgroup according to KDIGO is presented in Table 4.

**Figure 1:**
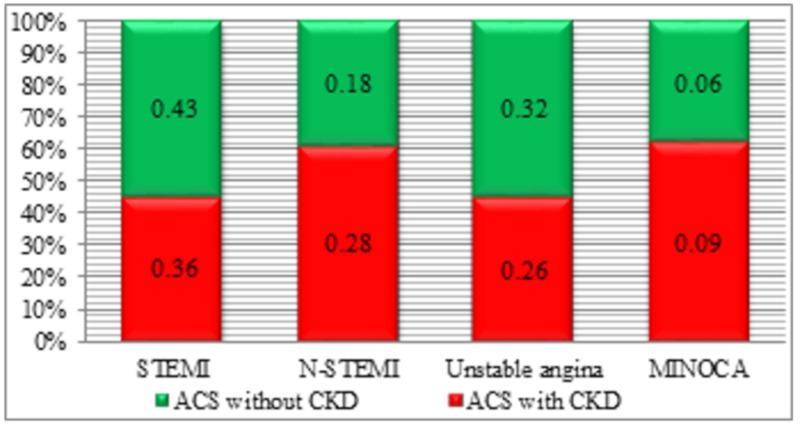
Acute coronary syndrome (ACS) categories in ACS with/without chronic kidney disease (CKD) groups.

The ACS repartition, considering the four main types, revealed an increased incidence of STEMI in the non-CKD group without statistical significance (*n* = 59 cases, *p* = 0.19), followed by the unstable angina in subjects with normal kidney function (*n* = 44 cases, *p* = 0.27) and NSTEMI in the CKD group (*n* = 39 cases) with statistical significance (*p* = 0.04), compared to the reported number in the non-CKD group (*n* = 25 cases). MINOCA had higher incidence in the CKD group (*n* = 13 cases) without statistical significance (*p* = 0.26), compared with the non-CKD group (*n* = 8 cases) (Figure 1). The possibility of a type 2 myocardial infarction secondary to anaemia was considered, but the argument for MINOCA was that, after the anaemia correction, the symptoms and changes in the electrocardiogram persisted. In the context of a type 2 myocardial infarction (secondary to a deficit between supply and demand for oxygen, once anaemia is corrected), there should be a remission of the symptoms and electrocardiographic changes.

The medical history of the study groups illustrated an increased percentage of previous chronic coronary syndrome and stable angina in the CKD group, with an important statistical value (*p* < 0.01) compared to the percentage reported in the non-CKD group. Previous heart failure was quantified through the NYHA classes and reflected a frequency of 41.61% in the CKD group, with statistical relevance (*p* < 0.01) compared to the non-CKD group (19.85%). Other comorbid conditions such as sequelae of myocardial infarction in the inferior territory (*p* = 0.01), previous ischemic stroke (*p* < 0.01), and diabetes mellitus (*p* < 0.01) were significantly different in the groups (Table 5). Symptomatology at presentation included thoracic pain, dyspnoea, and syncope. The specific thoracic pain showed a significant incidence (*p* < 0.01) in the non-CKD group, whereas dyspnoea was specific in the CKD group (*p* < 0.01). Loss of consciousness at admission was detected more rarely, without statistical importance (*p* = 0.16).

**Figure 2:**
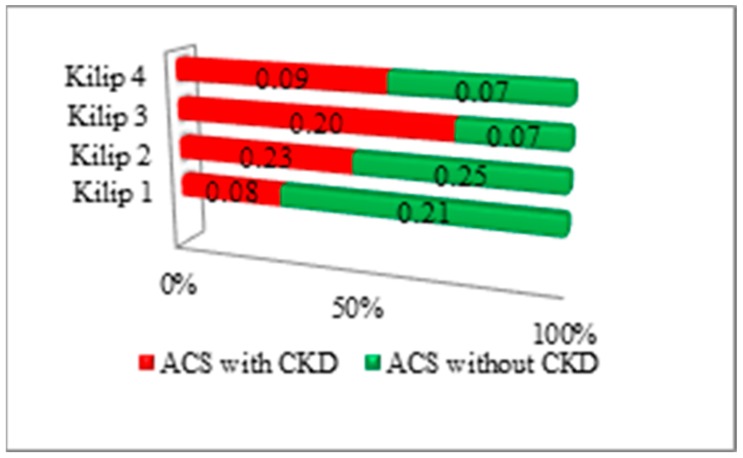
Killip–Kimball class assessment in subjects with ST-segment elevation myocardial infarction (STEMI) in both ACS with/without CKD subgroups.

The assessment of Killip class at admission in patients with STEMI demonstrated a high incidence (*p* < 0.01) of Killip 1 class in non-CKD group, and Killip 3 class had a significant incidence (*p* < 0.01) in the CKD group, as shown in Figure 2.

The incidence of cardiogenic shock in patients with ACS was higher in the CKD group without statistical relevance (*p* = 0.07). Left bundle branch block identified on the ECG was significant (*p* = 0.04) in the CKD group. New necrosis waves (Q wave) expressed statistical relevance (*p* < 0.01) in the non-CKD group. Electrocardiogram changes, presented at admission in both ACS with/without CKD subgroup, are shown in Table 6.

**Figure 3:**
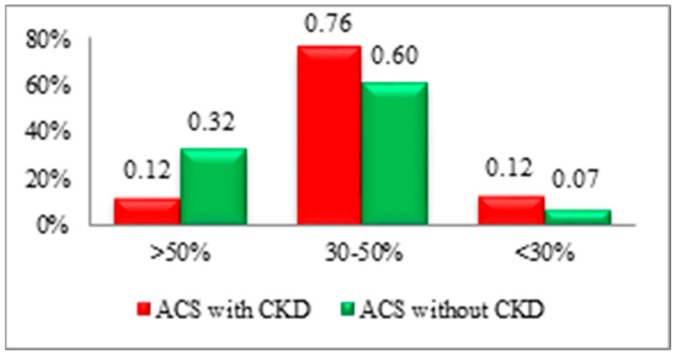
Ejection fraction (EF) of the left ventricular (LV) evaluated with Simpson’s method in both ACS with/without CKD subgroups.

The biomarker attesting the cardiac origin of dyspnoea (NT-proBNP) is a predictor of the prognosis for subjects diagnosed with ACS and CKD. The mean value of the marker was increased in subjects with impaired renal function (8259.67), standard deviation (Q1 = 930, Q3 = 12.495), thus explaining the significant incidence of acute pulmonary oedema and cardiogenic shock in these subjects. The group of patients with ACS and normal renal function showed lower mean values of the biomarker, with the rate of complications also being reduced (NT-proBNP = 4103.81, Q1 = 450.5, Q3 = 5550) (Figure 3).

The echocardiogram wall-motion abnormalities were characteristic in the CKD group with ACS. Normal LV contraction was found mostly in the non-CKD patients. Assessment of the regional LV wall motion abnormalities revealed significant elements such as dyskinesia of the basal segment of interventricular septum (*p* < 0.01) and akinesia of the basal segment of interventricular septum (*p* < 0.01) in the CKD group. Hypokinesia of the apical segment of the interventricular septum (*p* = 0.03) and akinesia of the middle portion of the interventricular septum (*p* < 0.01) were significant in the non-CKD group.

**Figure 4:**
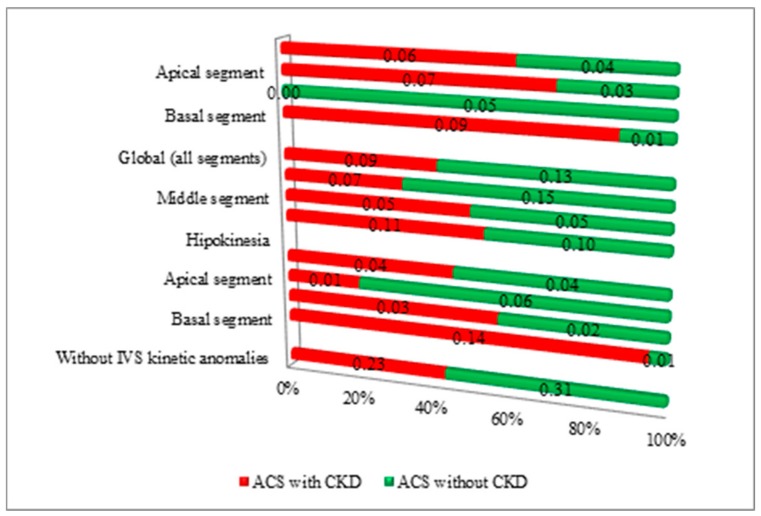
Disturbances of contractility in LV walls segments in both ACS with/without CKD subgroups.

Among the CKD group, coronary angiography was performed for 88.32% of the cases, whereas 99.26% of the cases from the non-CKD group underwent coronary angiography (*p* < 0.01). Medical management was selected for 11.68% of the cases from the CKD group, in contrast with the low rate of 0.74%, used in the non-CKD group. The disturbances of contractility in LV wall segments in both ACS with/without CKD subgroups are revealed in Figure 4. The analysis regarding the severity of CAD revealed that single-vessel CAD was specific in the non-CKD group (29.41%) showing statistical significance (*p* < 0.01) compared with the decreased incidence from the CKD group (10.95%). LMCA and three-vessel CAD were mostly detected in the CKD group, without statistical relevance (*p* = 0.13 and *p* = 0.42) compared to the non-CKD group (Table 7).

**Figure 5:**
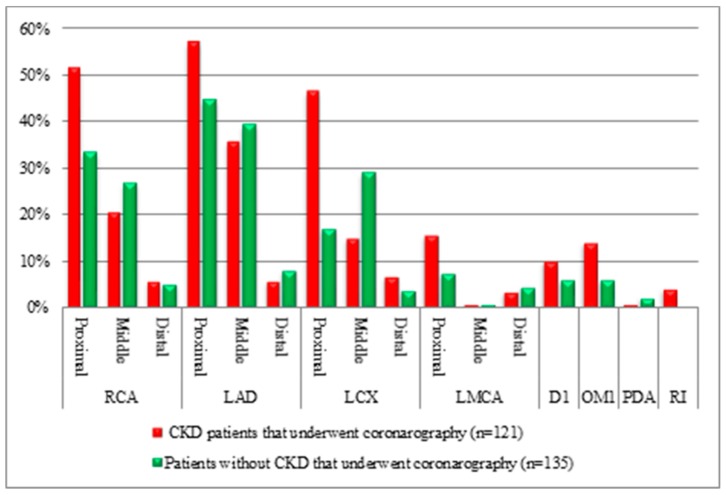
Significant coronary artery lesions in ACS subjects with/without CKD, revealed after coronary angiography. RCA—right coronary artery, LAD—left anterior descending artery, LCX—left circumflex artery, LMCA—left main coronary artery, D1—diagonal artery-first branch, OM1—obtuse marginal artery-first branch, PDA—posterior descending artery, RI—ramus intermedius branch.

The distribution of the coronary lesions illustrated that the proximal segments of the coronary arteries, including the right coronary artery (RCA), left anterior descending artery (LAD), and LMCA, were statistically significant (*p* < 0.01, *p* < 0.01, *p* = 0.03) in the CKD group. In addition, the obtuse marginal branch and the intermediate branch were mostly affected in the CKD subjects, with statistical significance (*p* = 0.02, *p* = 0.01) compared to the non-CKD group (Figure 5).

In order to identify if the biomarker values change within the two groups, a Mann–Whitney test was applied; statistically significant differences were obtained in the GFR, creatinine, urea, uric acid, NT-proBNP, CRP, and Fe cases (*p* < 0.001). In the case of cardiac troponin I (cTnI), a value of *p* = 0.226 was obtained; in the case of high sensitive troponin I (hs-TnI), the *p*-value was 0.120; in the case of the number of coronary arteries affected in CAD, *p* = 0.083. Thus, for these three cases, there were no statistically significant differences (*p* > 0.05). 

At the end of the study, only the study group was analysed in order to identify any associations between the biomarker values (GFR, creatinine, urea, uric acid, cTnI, hs-cTnI, NT-proBNP, CRP) and both the EF and the number of coronary arteries affected. Therefore, a multilinear analysis was applied, and the following results were observed:

- for the association between the GFR, creatinine, urea, uric acid, cTnI, hs-cTnI, NT-proBNP, CRP, and the EF values, a positive significant medium correlation resulted (*r* = 0.651, *R*^2^ = 0.424, *p* < 0.001);
- for the second case, where the association between GFR, creatinine, urea, uric acid, cTnI, hs-cTnI, NT-proBNP, CRP, and the number of coronary arteries affected in CAD were tested, a positive significant weak correlation was obtained (*r* = 0.377, *R*^2^ = 0.142, *p* = 0.011).

## 4. Discussion

This study showed that symptomatology in ACS is usually atypical and thoracic pain is predominantly described in the case of subjects with normal kidney function. Dyspnoea expressed statistical significance in CKD subjects, and studies revealed the fact that silent ischemia or atypical symptomatology is more frequently observed on admission in patients with impaired renal function. In a trial which enrolled 356 patients, painless myocardial infarction had an increased incidence in the CKD group versus the non-CKD subjects with statistical relevance [25]. The possible mechanisms responsible for the aforementioned phenomenon are diabetic neuropathy, a common pathology among CKD subjects, and neuropathy induced by uremic milieu [25].

ACS presenting dyspnoea as the main symptom can be mistaken for angina equivalent. Sosnov et al. reported that subjects with CKD were 43% less likely to describe specific thoracic pain in comparison with the subjects without CKD, regardless of the presence of diabetic neuropathy. This observation is similar to the present study, which revealed an important incidence of shortness of breath in the CKD subgroup [26].

The paucisymptomatic clinical picture and the anginal equivalences characteristic to ACS, highlighted in the case of subjects with CKD, are essential elements in the elaboration of a rigorous screening procedure [27]. The role of cardiovascular risk factors, independent of renal function, has been highlighted by numerous studies [28,29], the most significant being that performed by Go et al. [30] which included subjects with CKD. The mentioned research was able to illustrate that the impairment of renal function significantly influences the rate of hospitalization in the case of ischemic CAD, obliterative CAD (peripheral artery disease—PAD), or congestive heart failure, regardless of the associated cardiovascular risk factors, highlighting the role of independent predictor of the major cardiovascular events [30]. The same associations and similar prognosis have been demonstrated in subjects diagnosed with ACS and CKD, even after eliminating the major common cardiovascular risk factors. Results of the global registry of acute coronary events study (GRACE study−which included 11,774 subjects diagnosed with ACS) independently associated creatinine clearance with mortality, especially in patients with severe CKD even after adjusting the influence of other cardiovascular risk factors [31].

Electrocardiogram findings consistent with ischemia are not characteristic in CKD patients, because these subjects usually have left ventricular hypertrophy (LVH) or left bundle branch block. The present study revealed that left bundle branch block and QS waves are mostly present in the CKD subjects being statistically relevant (*p* < 0.01).

A retrospective study including patients from two important registries, the National Institute of Diabetes and Digestive and Kidney Diseases and the Third National Registry of Myocardial Infarction (NRMI-3), demonstrated that left bundle branch block incidence was higher in the advanced CKD group compared to the other CKD subjects [32].

The different evolution of ACS in CKD and normal renal function is explained by the particularities of the atherosclerotic process in the uremic environment. Thus, the interaction of classic and non-traditional cardiovascular risk factors will generate a chronic inflammatory process, also materialized by increased CRP values in CKD subjects. The chronic inflammation will be the substrate of endothelial dysfunction. Ventricular remodelling occurring after the acute coronary event is more common in subjects with CKD. The clinical study published by Naito et al. [33] illustrated that patients with mild or moderate CKD have a poor prognosis after ACS compared with subjects with normal renal function. Increased levels of CRP and interleukin-6 at hospital admission, in conjunction with increased BNP (brain natriuretic peptide) biomarker in convalescence, reflect the association between ventricular remodelling after myocardial infarction and impaired renal function. Chronic inflammation and oxidative stress have an increased incidence in CKD and are the main elements responsible for the onset of the aforementioned complication. It has also been shown that CKD is an independent predictor of ventricular remodelling occurrence after ACS [33]. Therapeutic classes that prevent ventricular remodelling (such as angiotensin-converting enzyme inhibitors and mineralocorticoid receptor antagonists) are indicated in subjects with ACS and CKD (who also have predictors of ventricular remodelling). Moreover, in addition to periodic echocardiographic evaluation of ventricular cavities, the therapeutic regimen should be reviewed frequently, especially in CKD subjects, with the occurrence of severe hyperkalemia being possible.

Herzog et al. conducted a study that compared the characteristics of 3049 dialyzed patients with those of 534,395 non-CKD subjects [34]. The results emphasized that dialyzed patients have a higher Killip class at admission in contrast with the non-CKD subjects, especially Killip class II and III, described as presence of rales in less than a half of the thoracic field and pulmonary oedema, respectively [34]. The observations were similar to the present research findings.

A cross-sectional study in the chronic renal insufficiency cohort (CRIC study) evaluated the high-sensitivity troponin in subjects with impaired renal function without myocardial ischemia and revealed that a high-sensitivity troponin, more than 3 ng/L, correlates with the LV mass and impaired LV performance (EF < 40%). Increased values of the mentioned cardiac necrosis enzymes predict high incidence of heart failure in CKD subjects [35].

NT-proBNP negatively influences the prognosis of the CKD subgroup. Though the elevation of the biomarker is common in asymptomatic subjects with impaired renal function, one study revealed that high values of NT-proBNP associated with a decreased GFR less than 60 mL/min/1.73 m^2^ can identify subjects with high mortality risk [36]. Thus, NT-proBNP remains an essential element in identifying high-risk patients and offers details regarding the outcome, conclusions similar with the findings of the present study.

CAD has several particularities in CKD consisting in high incidence, rapid progression, elevated prevalence of multi-vessel disease, and presence of vascular calcifications that interfere with proper stent expansion. Results regarding the high incidence of three-vessel CAD and LMCA in CKD patients were similar to other findings of similar studies, but they did not reach the statistically significant number due to the low number of patients and a single experimental centre [34,37,38,39]. Revascularization with PCI or CABG is challenging.

Another aspect is illustrated by lack of retrospective studies comparing the two myocardial revascularization methods, due to the exclusion of subjects with deteriorated renal function or inclusion of an irrelevant number of subjects. The existing observational studies are the only source of analyses regarding the benefits of both PCI and CABG in CKD patients. Chan et al. revealed that three-year major adverse cardiac and cerebrovascular events (MACCE) and survival were lower after the CABG versus the interventional myocardial revascularization using drug eluting stents (DES) in 893 subjects with CKD [40]. A larger study designed by Bangalore including 2960 subjects with CKD revealed that the only benefit of CABG versus PCI is represented by the decreased rate of repeated myocardial revascularization and myocardial infarction reoccurrence during a four-year period of follow-up [41]. Death was not significantly influenced by the chosen myocardial revascularization option. CABG was associated with a high incidence of short-term death and stroke [41]. A five-year analysis of the Syntax trial emphasized that subjects with CKD had higher rates of stroke and myocardial infarction compared to the patients with normal kidney function [16]. CABG was superior to PCI in subjects with CKD and diabetes mellitus regarding the five-year rates of MACCE and death [10]. The repeated revascularization was lower in the CABG group in contrast with the PCI group, with statistical importance [42].

The discrepancy between these trials is due to the different generation of DES used in the PCI groups. The Syntax trial reports usage of first-generation DES and Bangalore mentions usage of second-generation DES (paclitaxel eluting stents) with obvious superiority [41].

In this study, most of the patients diagnosed with STEMI and CKD benefited from the same therapeutic approach, adopted also in the subjects with normal renal function, namely, the interventional or surgical myocardial revascularization. There were seven patients who had STEMI and impaired renal function; they refused the coronary procedure, after the risks and benefits of the procedure were explained to them, being treated conservatively. Fibrinolysis was not performed in these patients, as they were not indicated for this procedure (the onset of angina symptomatology being at a time interval >12 h). In addition, conservative treatment was an option in eight of the cases with NSTEMI that refused the intervention or surgical revascularization.

Complications (such as acute pulmonary oedema or cardiogenic shock) were more frequently detected in the group of CKD patients, compared to the group of subjects with normal renal function. Severe CAD, EF impairment of left ventricle, ischemia of papillary muscles of the mitral valve, and secondary severe mitral valve insufficiency in conjunction with impaired renal function are responsible for the negative prognosis and complications commonly encountered in these patients.

The group of subjects with ACS and normal renal function showed a favourable evolution of ACS, with the rate of lung oedema and cardiogenic shock being lower. The results of the present research indicate that the management of patients with ACS and CKD in the emergency department should consider certain evolutionary features and the negative prognosis. Given the increased frequency of multivessel coronary disease and hemodynamic instability commonly encountered in CKD, special attention will be paid to echocardiographic examination aimed at identifying the severity of parietal kinetic disorders and the estimated EF. The evaluation will be performed at the presentation of the patient in the emergency service, without delaying the coronarography. The highly responsive NT-proBNP biomarker and severely impaired renal function are other useful predictors in identifying patients with ACS and CKD, likely to develop acute pulmonary oedema or cardiogenic shock. Additionally, the arrhythmogenic potential is considerable for the subjects with ACS and CKD, and the therapeutic target will focus on the early myocardial reperfusion and the correction of various dyselectrolytemias.

The above-mentioned observations outline the hypothesis that myocardial revascularization performed in the shortest period of time in CKD patients, even in the case of NSTEMI, improves the prognosis and reduces the risk of hemodynamic instability. The Sweetheart registry has shown that early myocardial reperfusion positively influences short-term survival in patients with mild or moderate CKD and NSTEMI [43].

## 5. Conclusions

CKD may be associated with CAD. The present research revealed that subjects with CKD with coronary events associate coexisting pathologic conditions such as diabetes mellitus, heart failure, previous ischemic stroke, and chronic CAD. Elements such as NT-proBNP and echocardiography should be included in the evaluation of these patients, considering the great contribution to establish the overall prognosis. Proximal and severe CAD are characteristic for subjects with deteriorated renal function. New trials including large numbers of subjects with CKD and ACS should be performed to evaluate the clinical characteristics and the evolution of the subjects, and a strategy to improve the outcome in CKD subjects with ACS should be developed.

## Figures and Tables

**Table 1:** The five stages of chronic kidney disease (CKD) described according to the glomerular filtration rate (GFR) decrease.

Stage	CKD	GFR (mL/min/1.73 m^2^)
1	Normal kidney function	≥90
2	Mild loss of kidney function	60–89
3	Moderate loss of kidney function	30–59
4	Severe loss of kidney function	15–29
5	Chronic dialysis treatment	≤15

**Table 2:** New York Heart Association (NYHA) classification of heart failure.

Class	The Severity of Shortness of Breath
I	No shortness of breath when performing ordinary activities
II	Slight limitations of activities due to the shortness of breath occurrence
III	Dyspnoea in most of the ordinary activities with marked limitation of the physical activities
IV	Shortness of breath at rest and inability to carry physical activities without symptoms occurrence

**Table 3:** Gender distribution in both acute coronary syndromes (ACS) with/without CKD subgroups.

Characteristics	ACS with CKD (*n* = 137)		ACS without CKD (*n* = 136)		*p* *
**Gender**	**No.**	**%**	**No.**	**%**	0.76
Men	93	67.88	90	66.18	
Women	44	32.12	46	33.82	
Age					
Mean age	68.62 ± 9.94		64.19 ± 10.68		<0.01
95% confidence level	(48.74; 88.5)		(42.83; 85.55)		

* The *p*-value was obtained by applying a Mann–Whitney test.

**Table 4:** Staging of the CKD in the ACS with CKD subgroup according to Kidney Disease: Improving Global Outcomes (KDIGO).

GFR	Patients	
	No.	%
Stage 3	89	64.96
Stage 4	32	23.36
Stage 5	16	11.68

**Table 5:** Associated comorbidities in both ACS with/without CKD subgroups and main symptomatology at admission in the study group.

Comorbidities/Symptoms at Presentation	ACS with CKD		ACS without CKD		*p* *
	No.	%	No.	%	
Chronic coronary syndrome	62	45.26	35	25.74	<0.01
Stable angina	54	39.42	35	25.74	<0.01
Canadian Cardiovascular Society grading of angina pectoris					
Degree I	8	5.84	6	4.41	0.59
Degree II	29	21.17	23	16.91	0.37
Degree III	17	12.41	6	4.41	0.01
Previous heart failure	57	41.61	27	19.85	<0.01
NYHA II	32	23.36	15	11.03	<0.01
NYHA III	25	18.25	12	8.82	0.02
Sequelae of myocardial infarction	30	21.90	15	11.03	0.01
Anterior territory	15	10.95	11	8.09	0.42
Inferior territory	14	10.22	3	2.21	<0.01
Lateral territory	0	0.00	1	0.74	0.31
Anterior and inferior territories	1	0.73	0	0.00	0.31
Peripheral artery disease	24	17.52	16	11.76	0.17
Previous ischemic stroke	25	18.25	2	1.47	<0.01
Diabetes mellitus	78	56.93	48	35.29	<0.01
Thoracic pain at admission	78	56.93	118	86.76	<0.01
Dyspnoea at admission	57	41.61	18	13.24	<0.01
Syncope at admission	2	1.46	0	0.00	0.16
Cardiogenic shock (%)	18	13.14	9	6.62	0.07

* The *p*-value was obtained by applying a chi-squared test for percentage.

**Table 6:** Electrocardiographic changes presented at admission in both ACS with/without CKD subgroup.

ECG at Admission	ACS with CKD		ACS without CKD		*p* *
	Patients				
	No.	%	No.	%	
ST-segment elevation	51	37.23	55	40.44	0.58
ST-segment depression	25	18.25	23	16.91	0.77
QS waves	2	1.46	13	9.56	<0.01
T negative waves	23	16.79	20	14.71	0.63
Left bundle branch block	18	13.14	8	5.88	0.04
Right bundle brunch block	7	5.11	5	3.68	0.56
Left ventricle hypertrophy	5	3.65	1	0.74	0.1
Ventricular paced rhythm	1	0.73	1	0.74	0.99
Complete atrioventricular block	1	0.73	0	0.00	0.31
No ECG changes	4	2.92	10	7.35	0.09

* The *p*-value was obtained by applying a chi-squared test for percentage.

**Table 7:** Coronary artery disease (CAD) characteristics in both ACS with/without CKD subgroups.

Characteristics	ACS/CKD		ACS		*p* *
	No.	%	No.	%	
Single-vessel CAD	15	10.95	40	29.41	<0.01
Double-vessel CAD	37	27.01	44	32.35	0.33
Three-vessel CAD	37	27.01	31	22.79	0.42
LMCA	20	14.60	12	8.82	0.13
MINOCA	12	8.76	8	5.88	0.36
Patients without coronary angioplasty	16	11.68	1	0.74	<0.01

* The *p*-value was obtained by applying a chi-squared test for percentage. CAD–coronary artery disease; LMCA–left main coronary artery; MINOCA–myocardial infarction with nonobstructive coronary arteries.
